# How can patient preferences be used and communicated in the regulatory evaluation of medicinal products? Findings and recommendations from IMI PREFER and call to action

**DOI:** 10.3389/fphar.2023.1192770

**Published:** 2023-08-16

**Authors:** Rosanne Janssens, Liese Barbier, Mireille Muller, Irina Cleemput, Isabelle Stoeckert, Chiara Whichello, Bennett Levitan, Tarek A. Hammad, Charis Girvalaki, Juan-Jose Ventura, Karin Schölin Bywall, Cathy Anne Pinto, Elise Schoefs, Eva G. Katz, Ulrik Kihlbom, Isabelle Huys

**Affiliations:** ^1^ Department of Pharmaceutical and Pharmacological Sciences, KU Leuven, Leuven, Belgium; ^2^ Novartis Pharma AG, Basel, Switzerland; ^3^ Belgian Healthcare Knowledge Centre (KCE), Brussels, Belgium; ^4^ Bayer AG, Wuppertal, Germany; ^5^ Evidera, London, United Kingdom; ^6^ Global Epidemiology, Janssen R&D, LLC, Pennsylvania, PA, United States; ^7^ Takeda Pharmaceuticals, Cambridge, MA, United States; ^8^ European Cancer Patient Coalition (ECPC), Brussels, Belgium; ^9^ School of Health, Care and Social Welfare, Division of Health and Welfare Technology, Mälardalen University, Västerås, Sweden; ^10^ Department of Public Health and Caring Sciences, Centre for Research Ethics & Bioethics, Uppsala University, Uppsala, Sweden; ^11^ Merck & Co., Inc., Rahway, NJ, United States; ^12^ Janssen Global Services, LLC, Raritan, NJ, United States; ^13^ Department of Learning, Informatics, Management and Ethics, Karolinska Institutet, Solna, Sweden

**Keywords:** patient preferences, patient experience data, regulatory evaluation, scientific advice, benefit-risk assessment, clinical trial endpoint selection, post-marketing assessment

## Abstract

**Objective:** Patients have unique insights and are (in-)directly affected by each decision taken throughout the life cycle of medicinal products. Patient preference studies (PPS) assess what matters most to patients, how much, and what trade-offs patients are willing to make. IMI PREFER was a six-year European public-private partnership under the Innovative Medicines Initiative that developed recommendations on how to assess and use PPS in medical product decision-making, including in the regulatory evaluation of medicinal products. This paper aims to summarize findings and recommendations from IMI PREFER regarding i) PPS applications in regulatory evaluation, ii) when and how to consult with regulators on PPS, iii) how to reflect PPS in regulatory communication and iv) barriers and open questions for PPS in regulatory decision-making.

**Methods:** PREFER performed six literature reviews, 143 interviews and eight focus group discussions with regulators, patient representatives, industry representatives, Health Technology Assessment bodies, payers, academics, and clincians between October 2016 and May 2022.

**Results:** i) With respect to PPS applications, prior to the conduct of clinical trials of medicinal products, PPS could inform regulators’ understanding of patients’ unmet needs and relevant endpoints during horizon scanning activities and scientific advice. During the evaluation of a marketing authorization application, PPS could inform: a) the assessment of whether a product meets an unmet need, b) whether patient-relevant clinical trial endpoints and outcomes were studied, c) the understanding of patient-relevant effect sizes and acceptable trade-offs, and d) the identification of key (un-)favorable effects and uncertainties. ii) With respect to consulting with regulators on PPS, PPS researchers should ideally have early discussions with regulators (e.g., during scientific advice) on the PPS design and research questions. iii) Regarding external PPS communication, PPS could be reflected in the assessment report and product information (e.g., the European Public Assessment Report and the Summary of Product Characteristics). iv) Barriers relevant to the use of PPS in regulatory evaluation include a lack of PPS use cases and demonstrated impact on regulatory decision-making, and need for (financial) incentives, guidance and quality criteria for implementing PPS results in regulatory decision-making. Open questions concerning regulatory PPS use include: a) should a product independent broad approach to the design of PPS be taken and/or a product-specific one, b) who should optimally be financing, designing, conducting, and coordinating PPS, c) when (within and/or outside clinical trials) to perform PPS, and d) how can PPS use best be operationalized in regulatory decisions.

**Conclusion:** PPS have high potential to inform regulators on key unmet needs, endpoints, benefits, and risks that matter most to patients and their acceptable trade-offs. Regulatory guidelines, templates and checklists, together with incentives are needed to foster structural and transparent PPS submission and evaluation in regulatory decision-making. More PPS case studies should be conducted and submitted for regulatory assessment to enable regulatory discussion and increase regulators’ experience with PPS implementation and communication in regulatory evaluations.

## 1 Introduction

Patients have unique, experience-based insights into their disease and treatments. As end-users of treatments, patients are or will be (in-)directly affected by each decision taken throughout the life cycle of these treatments (MEDICAL DEVICE INNOVATION CONSORTIUM MDIC, 2015; Muhlbacher and Kaczynski, 2016; Marsh et al., 2018). Prior research has revealed that the incorporation of patients’ views in decisions that affect them is not only warranted from an ethical point of view but may also improve the quality of decision-making and increase likelihood that decisions are supported by the patient population (van Til and Ijzerman, 2014; Danner et al., 2011; Egbrink and IJzerman, 2014; Hauber et al., 2013; Craig et al., 2017; Mott and Najafzadeh, 2016; Brooker et al., 2013; Dirksen, 2014; Mühlbacher and Johnson, 2017; The patient’s voice in the evaluation of medicines, 2013). One key stage in the process of bringing a treatment to market and the patient is the regulatory evaluation, where a medicinal products’ benefits are balanced against its risks and uncertainties.

In Europe, the European Medicines Agency (EMA), which is the central responsible regulatory body to authorise and monitor medicines in the European Union (EU), involves patients and consumers in different ways: i) having patients as members in its Management Board and some scientific committees, ii) consulting patients on specific requests by the scientific committees and working parties, iii) asking patients to review written information on medicines, and iv) involving patients in the prepation of regulatory guidelines and in the Agency’s conferences and workshops (European Medicines Agency, 2022a). In the US, the US Food and Drug Administration (FDA) is the regulatory authority responsible for the evaluation of human and veterinary drugs, biological products, and medical devices, and has set up a patient-focused drug development initiative. This initiative consists of public meetings that aim to obtain the patient’s perspective on specific diseases and treatments. In addition, the FDA also hosts externally led (informal) meetings in selected disease areas that allow patient organizations, patients, and caregivers to discuss their disease and symptoms (US Food and Drug Admini stration, 2020). Both EU and US regulators also increasingly encourage the use of Patient Reported Outcomes Measures (PROM)s. Patient Reported Outcomes (PROs), elicited via PROMs, provide important information on treatment effects and are increasingly included as endpoints in clinical trials (Teixeira et al., 2022). This enables patients to report their experience with regards to generic health or disease outcomes as well as disease specific burdens and treatment side-effects (US Food and Drug Administration, 2021; European Medicines Agency, 2022b; Teixeira et al., 2022). While the patient engagement methods that are currently most applied by regulators (patient consultation and PROMs), aim to understand and assess concepts that are of importance to patients, they typically do not provide (relative) weights for different health and disease outcomes or other treatment attributes that allow for trade-off calculations. The use of patient preferences assessed via patient preference studies (PPS), which can be used to serve this exact purpose, is therefore increasingly being recognized as a highly complementary method to inform decision-making regarding treatments throughout their entire life cycle, from development to use in clinical practice (The PREFER consortium, 2022), including during regulatory evaluation and decision-making. PPS provide qualitative and quantitative evidence from patients on the relative importance of what matters to patients and provide information on patients’ views about the acceptability of trade-offs between treatment characteristics, or other attributes of treatments or health interventions.

Stakeholder research has shown the potential value and importance of incorporating patient preferences considering that the views of the end user are crucial (van Overbeeke et al., 2019a; Whichello et al., 2019; Janssens et al., 2019a). Regulators in particular have expressed that the use of patient preferences could help improve their decision-making as it adds unique views and experiences related to both the disease and the treatment (effects) being evaluated (Janssens et al., 2019a; van Overbeeke et al., 2019a; Whichello et al., 2019). Patients often value and weigh aspects differently than regulators (or clinicians) and could indicate as such the importance of certain disease or treatment aspects that otherwise could be overlooked (Janssens et al., 2019a; van Overbeeke et al., 2019a; Whichello et al., 2019). Insights regarding what effects and endpoints a patient would find to be important to them would be difficult or impossible to know from a purely regulatory viewpoint and could therefore add or even change regulators’ overall views and conclusions (Janssens et al., 2019a; van Overbeeke et al., 2019a; Whichello et al., 2019). Concretely, patient preferences could support regulatory assessment by improving the understanding of i) the disease burden and unmet needs, ii) the relevance of primary and secondary endpoints to patients (Janssens et al., 2019a), iii) the relevance of specific side effects, and iv) the importance of potential improvements claimed by the applicant (Janssens et al., 2019a; van Overbeeke et al., 2019a; Whichello et al., 2019). To this end, PPS need to be robust, methodologically strong, transparently reported, and well considered as a condition to be valuable to inform decision-making (Janssens et al., 2019a; van Overbeeke et al., 2019a; Whichello et al., 2019).

In this way, PPS can complement the aforementioned methods (direct engagement with individual patients or patient associations, and PROMs) that capture patients’ views and experiences useful in regulatory decision-making. The term Patient Experience Data (PED) is increasingly used by both the EMA and FDA as an umbrella term that includes these different types of patient data including PROs, direct patient engagement, patient preferences, and other relevant patient data (US Food and Drug Administration, 2021; European Medicines Agency, 2022b). An overview of definitions and types of PED is provided in Table 1.

**TABLE 1:** Terminology: patient experience data and related definitions.

Patient Experience Data (PED)	Umbrella term referring to “*data that are collected via a variety of patient engagement actitivities and methodologies to collect patients’ experiences of their health status, symptoms, disease course, treatment preferences, quality of life and impact of healthcare”.*(European Medicines Agency, 2022b)
	This includes patient engagement data, patient preferences, and patient reported outcomes.
Patient Engagement (PE)	Term that refers to *“all activities involving interaction with patients to gather their experience on disease, preferences, outcomes and treatments.''* (European Medicines Agency, 2022b).
Patient Preferences (PPs)	Term that refers to “*how desirable or acceptable is to patients a given alternative or choice among all the outcomes of a given medicine''* (European Medicines Agency, 2022b).
	Patient preferences are elicited via patient preference studies (PPS), which use qualitative and/or quantitative techniques such as stakeholder interviews (qualitative) and discrete choice experiments and questionnaires (quantitative) to assess the relative desirability or acceptability to patient of specified alternatives or choices among outcomes or other attributes that differ among alternative health interventions.
Patient Reported Outcomes (PROs)	Term that refers to “*a health/treatment outcome reported directly by the patient without the interpretation of a clinician or another person*'' (European Medicines Agency, 2022b).
	Patient reported outcomes are elicited via patient reported outcome measures (PROMs). PROMs are questionnaire instruments that can be generic or disease-specific (Churruca et al., 2021).
Patient Experience Evidence (PEE)	A term which is used when “*patient experience data qualified as valid scientific evidence following a scientific assessment''.* (European Medicines Agency, 2022b)
	*“Both PED and PEE are relevant and can complement each other for regulatory purposes; patient data is needed to generate evidence of meaningful outcomes for patients.''* (European Medicines Agency, 2022b)

PPS, patient preference studies; patient reported outcome measures (PROMs).

Recent regulatory advances in the realm of PPS include the identification of PPS as a key area in EMA’s regulatory science strategy for 2025 (European Medicines Agency, 2023a). Besides this, the EMA together with the International Council for Harmonization of Technical Requirements for Pharmaceuticals for Human Use (ICH), has published a reflection paper supporting opportunities for the development of new ICH guidelines to foster a globally harmonized approach for the inclusion of patient preferences in a way that is methodologically sound and sustainable for both regulated industry and regulatory authorities (The International Council for Harmonisation of Technical Requirements for Pharmaceuticals for Human Use ICH, 2020). Thus far, there are only a few published examples on the use of patient preferences in regulatory decisions, and these have mainly come from the US (3% approvals used a PPS between 2017–2020) with very few examples from the European Union (EU) (The PREFER consortium, 2022). For example, in the US, the FDA recently approved a tympanostomy delivery system in which a PPS determined the performance threshold to use as the primary endpoint in the pivotal clinical trial for the procedure (The PREFER consortium, 2022).

In 2016, a six-year European public-private partnership collaboration under the Innovative Medicines Initiative (IMI), called “PREFER”, was initiated. The IMI PREFER project aimed to develop recommendations on how to assess and use PPS in medical product decision-making, including in the regulatory evaluation of medicinal products. To this end, IMI PREFER undertook a series of qualitative research studies to examine stakeholder views towards PPS and conducted patient preference case studies to assess the usefulness of different patient preference methods in different disease domains (The PREFER consortium, 2022).

One of the key outcomes of the IMI PREFER project was the development of a PPS framework and recommendations for PPS design, conduct, analysis, and interpretation (The PREFER consortium, 2022). This PPS framework has been assessed by the EMA and the European network for Health Technology Assessment (EUnetHTA), and has received a positive qualification opinion in April of 2022 by EMA’s Committee for Medicinal Products for Human Use (CHMP) (European Medicines Agency, 2021a; European Medicines Agency, 2021b; IMI PREFER, 2021). This qualification opinion highlights the regulatory acceptability of the use of PPS in the development and assessment of medicinal products (European Medicines Agency, 2020; European Medicines Agency, 2021a). More concretely, the PREFER framework, which was assessed in the EMA qualification, consists of three components, and aims to inform PPS research teams on key considerations when designing, conducting, and applying the results of a fit-for-purpose PPS, and guide decision-makers when assessing and using PPS results to inform medical product decision-making. Over the past years, also the FDA has taken a pro-active position in outlining its interest in PPS use in treatment development and evaluation, such as via the development of guidance documents and organization of public workshops on PPS usage in regulatory environment (Ho et al., 2015a; Patient-Focused Drug Development, 2018; US Food and Drug Administration, 2020a; US Food and Drug Administration, 2020b; US Food and Drug Administration, 2022). The FDA’s recent draft guidance on benefit-risk includes, for example, considerations for PED and PPS in benefit-risk assessments (Food and Drug Administration, 2021).

Despite these positive steps forward from regulators, there is a need for more information and evidence-based insights towards all stakeholders involved in and affected by regulatory decision-making (including drug developers, researchers, regulators, Health Technology Assessment (HTA) bodies, and patients) on how PPS results can be used and communicated in regulatory evaluation and decision-making (Table 2). Such clarity is important to steer the design and incentivize conduct of PPS and thereby, R&D efforts to areas of unmet medical needs and the selection of endpoints in clinical testing that matter most to patients.

**TABLE 2:** Rationale and potential implications of this paper. *IMI, Innovative Medicines Initiative; PPS, patient preference studies*.

What is already known on this topic?	Patient preference studies (PPS) could help regulators understand patients’ perspectives on the importance of treatment outcomes and the benefit-risk tradeoffs patients are willing to make. However, PPS currently do not systematically inform regulatory decisions and despite many positive steps forward from regulators, no EU regulatory guidelines outlining their concrete application(s) in regulatory decisions exist. IMI PREFER was a six-year European public-private partnership under the Innovative medicines initiative that developed recommendations on how to assess and use PPS in medical product decision-making, including in the regulatory evaluation of medicinal products. The end-result of PREFER included recommendations intended to support development of (regulatory) guidelines on PPS.
What does this paper add?	This paper summarizes key findings and recommendations from IMI PREFER on how PPS can be concretely used and communicated in regulatory evaluation of medicinal products, including early decision-making (on unmet needs and relevant endpoints during horizon scanning activities and scientific advice), during regulatory evaluation (on patient acceptable benefit-risk trade-offs and relevant effect sizes) and in post-marketing safety assessments (during reassessment of product performance on patient-relevant endpoints and regulatory decision about continued approval). It highlights when and how stakeholders interested in conducting and submitting a preference study for regulatory evaluation can interact with regulators on PPS, including how they can make use of scientific advice procedures, how they can prepare for such interactions, and which topics and questions they should transparently report when communicating with regulators on PPS. It discusses future needed actions in terms of PPS implementation in regulatory documents, and outlines topics and open questions that should be addressed in regulatory guidelines on patient preferences.
How might this paper affect research, practice and policy?	Concrete applications for PPS usage presented in this paper could foster multi-stakeholder discussion and inform regulatory guidelines on the desired context(s) of use for PPS regulators consider fit-for-purpose in regulatory evaluation. Recommendations on when and how to consult with regulators may inform relevant discussion formats (such as scientific advice/joint consultations) on appropriate PPS research designs and may thereby lead to more robust and impactful PPS in regulatory decision-making. Barriers and open questions related to PPS usage in regulatory evaluation listed in this paper could trigger further discussion and critical reflection among the regulatory and broader stakeholder community and may foster the development of a policy agenda regarding future design and use of PPS.

The aim of this paper is to summarize findings and recommendations from the IMI PREFER project regarding patient preferences in regulatory evaluation and decision-making, and in particular:

• Applications for PPS in regulatory evaluation;
• When and how to consult with regulators to foster implementation of PPS in the regulatory evaluation process;
• How PPS could be reflected in regulatory documents and communication;
• Barriers and open questions for the use of PPS in regulatory decision-making.

## 2 Methods

**FIGURE 1:**
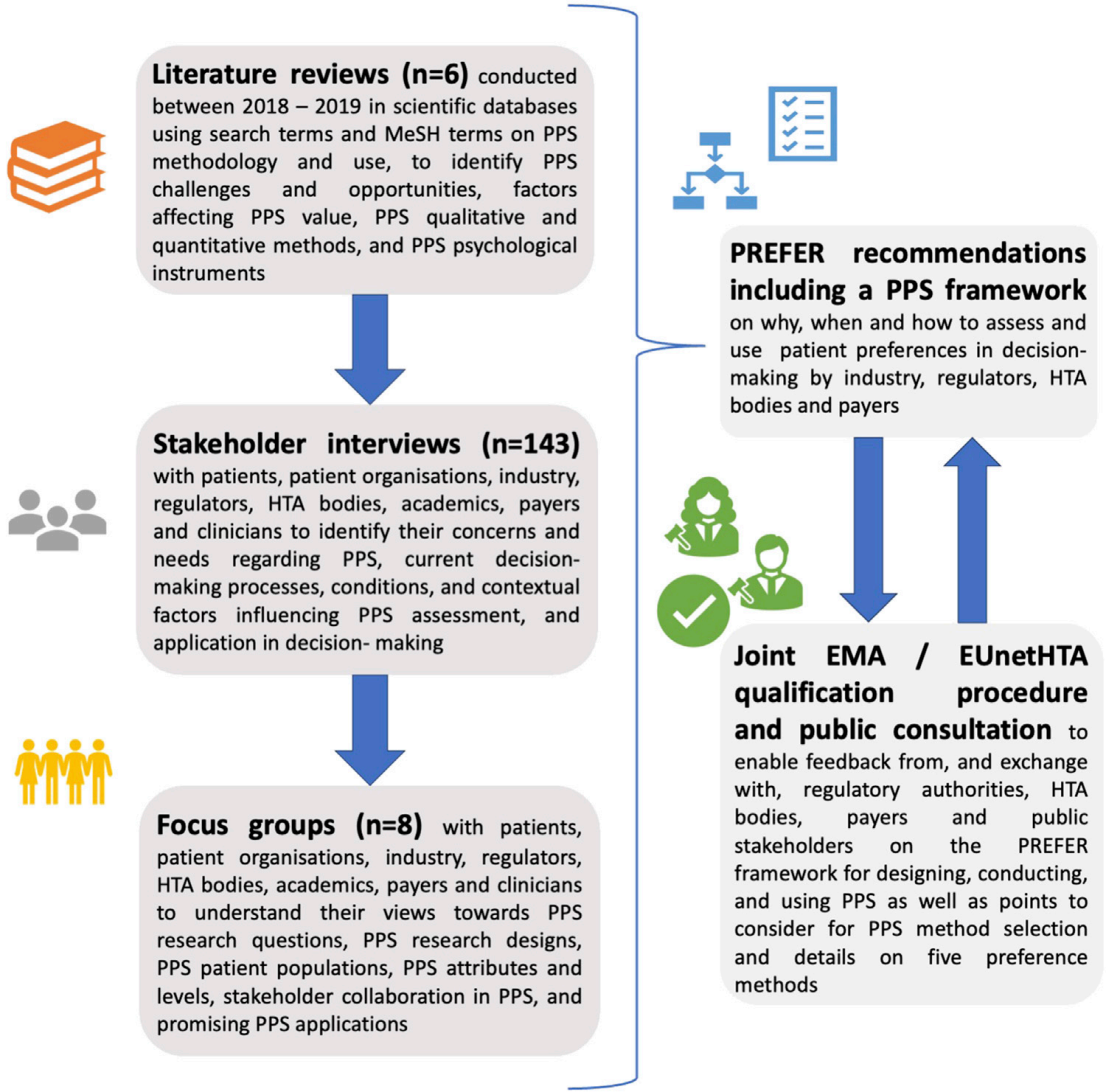
Research design used by PREFER to develop recommendations on how to assess and use patient preferences in medical product decision-making, including in the regulatory evaluation of medicinal products. Six (systematic/scoping) literature reviews, 143 individual stakeholder interviews and eight focus group discussions were held to inform the PREFER recommendations, including a framework for designing, conducting, and using patient preference studies as well as points to consider for method selection and details on five preference methods. Alongside the PREFER recommendations, PREFER undertook a parallel EMA/EUnetHTA Qualification Opinion procedure, which allowed PREFER to exchange with regulatory authorities, HTA bodies, and payers on the recommendations and enabled their input to be incorporated into the recommendations. As part of the methods qualification procedure, the EMA undertook a public consultation to obtain input on the PPS recommendations and framework. PPS, patient preferences studies; EMA, European Medicines Agency; EUnetHTA, European Network for Health Technology Assessment; n, number; HTA, Health Technology Assessment.

IMI PREFER adopted a mixed-methods stakeholder driven research design to develop recommendations relevant to the use of PPS results in the regulatory context. In particular, the research activities conducted within IMI PREFER that directly informed the recommendations included six **literature reviews** (Janssens et al., 2019b; van Overbeeke et al., 2019b; Russo et al., 2019; Soekhai et al., 2019; Whichello et al., 2020a; Whichello et al., 2020b), 143 **individual interviews** (Janssens et al., 2019a; Whichello et al., 2019) and eight **focus group discussions** (van Overbeeke et al., 2019a) with patients, patient organizations, academics, regulators, industry representatives, HTA bodies, payers, and clinicians (Figure 1). This research served to obtain an in-depth understanding towards PPS methodology and use in the context of regulatory evaluation. The interviews and focus group discussions were held with stakeholders in the EU and US between October 2016 and May 2022. This paper specifically describes findings relevant to the regulatory context as revealed by the perspectives of the various stakeholders (i.e., regulators, patient representatives, pharmaceutical industry representatives, academics, HTA bodies, payers, and clinicians) on this topic.

### 2.1 Scientific and grey literature reviews

The literature reviews (*n* = 6) (Janssens et al., 2019b; van Overbeeke et al., 2019b; Russo et al., 2019; Soekhai et al., 2019; Whichello et al., 2020a; Whichello et al., 2020b) aimed to understand current decision-making frameworks, including regulatory evaluation, and how results from PPS may be used within these. Search queries were developed based upon the key concepts of the review questions pertaining to the particular review, and consisted of free text words and Medical Subject Headings (MeSH) terms (for PubMed) and Emtree terms (for Embase) whenever available (e.g., relating to patient preferences “patient preference”[Mesh], the stakeholders who would use PPS results such as “drug industry”[MesH] and the decision-making context PPS could be used in e.g., “risk assessment”[MeSH]). Selected literature sources included systematic literature reviews, empirical qualitative and quantitative studies published in PubMed, Embase, EconLit, Guidelines International Network, PsycINFO and GoogleScholar databases outlining potential roles for preference studies across the treatment life cycle, regulatory strategies and guideline work relevant to PPS, websites of the EMA, ICH, and FDA, and reflection papers published by these authorities outlining their decision-making processes (i.e., grey literature) (The International Council for Harmonisation of Technical Requirements for Pharmaceuticals for Human Use ICH, 2020; Patient-Focused Drug Development, 2018; U.S. Department of Health and Human Services Food and Drug Administration CDRH Center for Biologics Evaluation and Research, 2015). Additionally, past and ongoing research projects about relating to how patient preferences can be used in (regulatory) decision making were reviewed (Institute for Quality and Efficiency in Health Care, 2014; Postmus et al., 2016; US Food and Drug Administration, 2016; Mühlbacher et al., 2017; Patient-Focused Drug Development, 2018; Postmus et al., 2018; Cook et al., 2019; Bridges et al., 2023). To further elucidate which potential PPS uses are of particular interest to stakeholders and in particular to regulators, the literature review covered the following: i) opportunities, challenges, and potential roles these stakeholders reported (E.g. Janssens et al., 2019a; van Overbeeke et al., 2019a; Janssens et al., 2019c), ii) the type of decisions during which preference studies would be most valuable for these stakeholders (van Overbeeke et al., 2019b; Whichello et al., 2019; Whichello et al., 2020a), and iii) preference study roles described by drug developers, regulators and other decision-makers (E.g. Ho et al., 2015b; US Food and Drug Administration, 2016; Bouvy et al., 2020). Inclusion and exclusion criteria were developed separately for each of the six literature reviews. Inclusion criteria related for example, to the literature types [e.g., regulatory documents, HTA reports, project reports and workshop reports (grey literature), (systematic) reviews, original research articles (e.g., published PP studies), and perspective articles (white literature)], or the use of a preference method in decision-making. Exclusion criteria for example, related to the language used, whether full text was available, and the publication date. The literature studies were conducted between 2016 and a final search for the data sources was performed in March 2023.

### 2.2 Stakeholder interviews and focus group discussions

The stakeholder interviews (*n* = 143) and focus group discussions (*n* = 8) (Janssens et al., 2019a; van Overbeeke et al., 2019a; Whichello et al., 2019) aimed to complement and gain further in-depth insights into the views of stakeholders who are involved in or affected by potential PPS use in regulatory treatment decision-making including EU (EMA) and US (FDA) regulators, patients, HTA bodies, payers, patient organizations, clinicians, academics, and pharmaceutical industry representatives (143 interviews and eight focus group discussions). Since the different stakeholders involved may have converging and diverging interests with respect to PPS, and the knowledge regarding PPS situates itself within a rapidly evolving time-context and changing mindset, the views of these different stakeholders were investigated. Furthermore, documentation developed as part of the PREFER project, material and feedback obtained during various multi-stakeholder discussion fora (between academic researchers, pharmaceutical company representatives, patient organization representatives, regulators, and payers) relating to the PREFER project informed the list of regulatory applications (e.g., DIA/IMI PREFER, 2023).

### 2.3 Joint qualification procedure with the European Medicines Agency and the European network for Health Technology Assessment

Finally, to further gain insights from regulators and HTA representatives on the impact and implementation of PPS in regulatory evaluation and beyond, PREFER initiated a joint qualification procedure with the EMA and EUNetHTA, the European Network for Health Technology Assessment (The PREFER consortium, 2022). The EMA accepted the application, and the full Qualification Opinion by the Committee for Medicinal Products for Human Use (CHMP) along with public comments is available on the EMA website (European Medicines Agency, 2021b; European Medicines Agency, 2021c; European Medicines Agency, 2022c). The value, process, and outcomes of this Qualification procedure and opinion are described in a separate paper (Müller et al., 2023).

## 3 Results

### 3.1 What are concrete applications for patient preference studies in regulatory decision-making?

#### 3.1.1 Before clinical trials, patient preference studies can inform decisions on unmet needs and clinical trial endpoints

**FIGURE 2:**
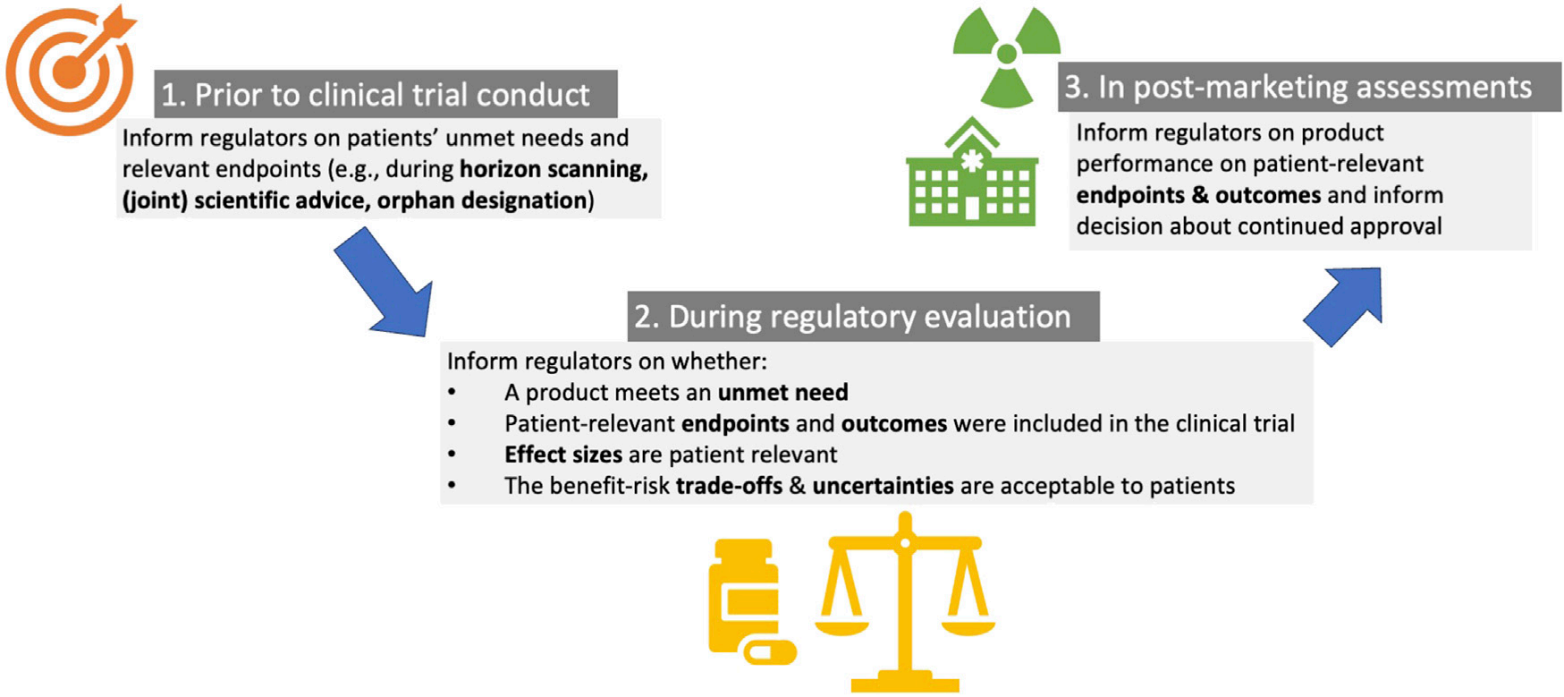
Applications for patient preference studies in regulatory evaluation of medicinal products, including in early decision-making prior to clinical trial conduct, during regulatory evaluation and in post-marketing regulatory assessments.

The PREFER studies highlighted how a PPS can, prior to the conduct of clinical trials, help regulators understand areas of unmet needs and where there is a need for treatment development (Figure 2). PPS evidence can inform the selection of primary and key secondary endpoints in the pivotal submission study, and thereby inform the selection of key benefits in the regulatory benefit–risk assessment (of note: a regulatory agency’s choice of key benefits will often be based on the primary and key secondary endpoints in the pivotal clinical trial) (Janssens et al., 2019a; van Overbeeke et al., 2019a; Whichello et al., 2019): stakeholders argued regulators could use PPS information on unmet medical needs when identifying potential high-impact products during their horizon scanning activities and help regulators understand whether there are products being developed that target such unmet needs. It was deemed that during scientific advice (early consultation) on clinical trial protocols, consideration on patient views of the relevance of the endpoints could be useful to include. This would allow to verify whether a particular clinical trial protocol developed by industry includes endpoints relevant to the patients as revealed in the PPS. Stakeholders noted how preferably, the view of different stakeholders should be aligned with the patient perspective in mind. In the EU, stakeholders noted this could be achieved through Joint EMA/EUnetHTA scientific consultations on clinical program (previously parallel consultations–see further).

#### 3.1.2 During regulatory evaluation of marketing authorization applications, patient preference studies can inform the evaluation of endpoints, key effects and uncertainties to consider in the benefit-risk assessment

PREFER revealed that PPS results can inform the regulatory approval by: i) evaluating whether a product targets patients’ prioritized treatment outcomes and endpoints “*which are actually meaningful to patients” (regulatory interviewee) (Janssens et al., 2019a)* (PPS results could inform whether the selected primary and key secondary endpoints in the pivotal submission study were those most relevant to patients), and thereby inform the selection of key benefits in the regulatory benefit–risk assessment, ii) understanding which trade-offs patients are (un-)willing to make between benefits and risks (i.e., wether patients would accept the side-effects in return for the benefits: “*would it be okay for them to have these side-effects provided when they get this effect?” (regulatory interviewee) (Janssens et al., 2019a)*, iii) understanding patient-relevant effect sizes “*I would like to ask them ‘we have this product, it has this effect, would that be relevant for you, or would it be just nothing” (regulatory interviewee) (Janssens et al., 2019a).* If evidence from PPS is available on what effect sizes are meaningful to patients, regulators can assess whether or not the product provides such a meaningful benefit; PPS may thereby provide a benchmark for the assessment of the relevance of the observed clinical data (does the new treatment offer a meaningful difference relative to the current standard of care?): “*You already have to make a value judgement, and you cannot make these kind of value judgement without having the preference of the patient involved in this decision-making process” (regulatory interviewee) (Janssens et al., 2019a)*, iv) identify key (un-)favorable effects and uncertainties (e.g., if the product causes side-effects that were perceived as detrimental; these problems could be included in the effects table).

#### 3.1.3 In post-marketing assessments, patient preference studies can inform the regulatory choice of endpoints and health outcomes to consider in case of post-launch re-assessment

The PREFER recommendations highlight that PPS could inform the reassessment of product performance on patient-relevant endpoints by regulators and regulatory decision about continued approval. At the post-marketing stage, the most relevant PPS were hence considered to be those that can inform acceptability of trade-offs between treatment characteristics. For example, the assessment of a rare but serious safety signal observed post-approval might gain from a PPS to understand how much risk patients are willing to accept in exchange for how much benefit.

### 3.2 When and how to consult with regulators to foster implementation of patient preference studies in the regulatory evaluation process?

IMI PREFER formulated the following recommendations with respect to when and how to consult with regulators to foster implementation of PPS in the regulatory evaluation process (The PREFER consortium, 2022):

1. PPS researchers are encouraged to have discussions with regulators before the start of the PPS to ensure that all relevant information will be captured in the PPS prior to its use in approval dossiers or product labels to inform regulatory decisions and/or assist in the interpretation of clinical study data generated for a specific product (The PREFER consortium, 2022). The cornerstone of early engagement with regulators is a scientific advice procedure. The purpose of scientific advice is to ensure that a PPS is designed such that its results are useful to the decision-maker. When patient preference research is expected to impact more than one decision-making process in the EU, multistakeholder alignment can be assisted by convergence mechanisms such as EUnetHTA-EMA joint scientific consultation. To maximise the benefits of scientific advice for PPS, it is recommended that (19):
    • All available guidance by regulatory and HTA bodies is considered. To complement this, and in the absence of detailed patient preference guidance, the IMI PREFER recommendations and EMA Qualification Opinion on IMI PREFER can be considered (The PREFER consortium, 2022);
  • Scientific advice procedures are initiated as early as possible when the study is intended to be used in decision-making by regulators (The PREFER consortium, 2022);
  • Regulators may want to include scientific experts in patient preference elicitation into the scientific advice processes. This is because preference studies use methodologies that are different from those used in clinical trials and observational studies and are more comparable to those used in utility elicitation studies. Protocol development advice requires expertise to assess the design and results of a preference study (The PREFER consortium, 2022);
2. Applicants, regulators, and HTA bodies should involve patients as research partners in the scientific advice process more frequently and systematically because their perspectives can complement the applicant’s scientific rationale for undertaking the patient preference study, and their views on the proposed design of the study should be taken into consideration (The PREFER consortium, 2022);
3. A PPS “briefing book” is advised to describe the PPS towards regulators. This PPS briefing book should consider available guidelines at the time of filing; the PREFER framework described in the PREFER recommendations could be used as basis where such guidelines do not exist or do not mention particular topics relevant for PPS (The PREFER consortium, 2022). The briefing book should optimally include the:
    • Rationale for conducting the PPS (e.g., lack of evidence regarding the trade-offs patients are willing to make between treatment characteristics)
  • Research question of the PPS and PPS study context;
  • PPS study methodology in sufficient depth, including the instrument to be used, the selection process of the instrument, and the instrument testing and revision process;
  • Ability of the patient preference study to quantify preference heterogeneity and allow estimation of trade-offs between treatment characteristics;
  • Internal and external validity of the research;
  • Involvement of patients as research partners and their contributions to date, as well as their future planned contributions (e.g., in data reporting and interpretation)
  • Limitations of the PPS, in context (The PREFER consortium, 2022).

The approach to undertake might be different depending on the nature of the PPS. For qualitative PPS, which are generally done as a first, exploratory step to gather in-depth insights from patients, PREFER formulated that they can be undertaken before obtaining scientific advice. In turn, for quantitative studies, advice is advised to be obtained before the study design is finalized and before start of data collection, so there is still opportunity to inform the design and set-up.

The CHMP Qualification Opinion describes that suitable topics for scientific advice are PPS sample definition, method selection, experiment/question design, and analysis, and that the focus of advisory interaction with regulatory bodies could be on the choice of the set of comparable alternatives (attribute vignettes), the range and presentation of attributes and their different levels (European Medicines Agency, 2021a; IMI PREFER, 2021).

### 3.3 In which regulatory documents can patient preference studies be included such that patient preference study usage can be externally communicated towards stakeholders?

#### 3.3.1 Patient preference studies can be included in the regulatory submission dossier submitted by the applicant

PPS intended for use in regulatory decision-making should be included in the submission dossiers by the applicant, providing the possibility for the regulator to assess this data as part of the overall dossier. In turn, patient preference results that were used to inform regulatory decision-making could be reflected in regulatory assessment reports, and in the product label (The PREFER consortium, 2022) (see below).

#### 3.3.2 Patient preference studies can be included in the regulatory assessment report

Including PPS data in external communication by the regulatory bodies would provide more transparency about how PPS have informed their decision-making and could help downstream decision-makers to inform their decision-making (e.g., HTA assessors, clinicians, patients).

The EMA Qualification Opinion states that *“In principle, information on PPS may be included in the Clinical Overview or the EPAR and other relevant documents. This would pertain to cases for which the information was either relevant to the regulatory decision and the benefit-risk assessment, and/or where PPS data are relevant to inform prescribers and users of the medicinal product. The decision will be made on a case-by-case basis.”* (European Medicines Agency, 2021a; IMI PREFER, 2021).

Indeed, when a PPS provides patients’ views about the most important *attributes* of a specific disease or medicinal product, PPS results would be useful to be reported in the EPAR, for example, in the section describing “*Available therapies and unmet medical need to support the description of unmet need*” (The PREFER consortium, 2022). Furthermore, results from such a PPS could inform the choice of endpoints included in the submitted clinical data package. These endpoints could have an influence on the choice of favourable effects included in the core summary, the “*Effects Table”* (The PREFER consortium, 2022).

For a preference study providing patients’ views about the *acceptability of a trade-off* between treatment characteristics or the acceptability of uncertainty, the preference study results could be included in the section describing “*Balance of benefits and risks*” and/or in the section describing “*Additional considerations on the benefit–risk balance*”. One further option would be the inclusion of such preference weights into the effects table in the section “Effects table” (The PREFER consortium, 2022).

Before inclusion in the EPAR, PPS results could already be part of the Day 80 Assessment Report. This would be consistent with the advice in the “EMA Day 80 Assessment report template about consideration of patient input” (The PREFER consortium, 2022). Three approaches for the evaluation of trade-offs between treatment characteristics are foreseen there: basic, descriptive, or quantitative assessment of trade-offs. Such an assessment in the EMA Day 80 report could include the three following aspects: i) a critical appraisal of the preference study, ii) the assessor’s view on the relative importance of the observed effects and/or view on maximum acceptable risk for a given level of benefit, informed by the results of the PPS, and iii) the extent to which this informs the assessor’s thinking on the acceptability of the benefit–risk assessment (The PREFER consortium, 2022). For a quantitative benefit–risk assessment, an EMA Day 80 report could include the three following aspects: i) a critical appraisal of the preference study, ii) the assessor’s view on the acceptability of the quantitative analysis in the “*Clinical Overview”*, and iii) the extent to which this informs the assessor’s thinking on the acceptability of the tradeoffs between treatment characteristics (The PREFER consortium, 2022).

#### 3.3.3 Patient preference studies can be included in the product labelling documents such as the summary of product characteristics

If PPS are included in the product label, this would provide information to patients and prescribers that could assist their decision-making in clinical practice.

For a regulatory decision where preference data played a key role in the approval of the product and/or may be of relevance for the prescriber and the patient when deciding on the prescription, PREFER proposes that preference data could be included in the “*Clinical efficacy and safety”* sub-section of Summary of Product Characteristics (SmPC) Section 5.1 “*Pharmacodynamic properties”*. (Alternatively, preference data could be included in a new “*Patient Experience”* sub-section of the SmPC Section 5.1.) (The PREFER consortium, 2022). In the Qualification Opinion, the CHMP notes that *“the value of conveying information on group-level preferences to individual patients in relevant documents would have to be carefully considered for situations where individual choice is paramount (i.e., for prescription or administration/use). If the primary intent was to reflect and justify the decision processes considered at the time of clinical program planning and during the marketing authorization assessment, the EPAR would appear a more appropriate place for PPS-related descriptions and/or data. As said, a final decision by CHMP would only be possible at the time of an assessment of a marketing authorization on a case-by-case basis, taking into account the validity and robustness of the data”.* (European Medicines Agency, 2021a; IMI PREFER, 2021).

Should it be appropriate to include preference results in the SmPC, this could be approached as follows:

• Summary of the situation prompting the preference study: For an “acceptability of trade-off” or “acceptability of uncertainty” scenario, a summary of the situation could be provided by describing the study purpose or the primary research question (The PREFER consortium, 2022);
• Description of the preference study design and population: This would be aligned with the approach typically taken in the description of clinical data, in which the SmPC template expects information on “the main characteristics of the patient population” (The PREFER consortium, 2022);
• Summary of the preference study results: This would also be aligned with the approach typically taken in the description of the clinical data, where the SmPC template expects this section to provide “evidence from relevant studies” (The PREFER consortium, 2022).

### 3.4 What are barriers and open questions for the use of patient preference studies in regulatory decision-making?

Stakeholders across groups raised several barriers with respect to PPS in regulatory and access evaluation; such as concerns regarding a lack of understanding on the definition and implementation of patient preferences *“we need to alphabetize the different stakeholders about what is the meaning of patient preferences and how we can use them meaningfully in order to have a better decision” (HTA interviewee)* (Janssens et al., 2019a; van Overbeeke et al., 2019a; Whichello et al., 2019). There was also called upon a lack of regulatory guidance and (financial) incentives for product developers and PPS researchers to invest in PPS (Janssens et al., 2019a; van Overbeeke et al., 2019a; Whichello et al., 2019). To move toward using PPS, stakeholders including regulators highlighted a need to: i) improve their understanding on preference methodologies and how PPS would benefit their assessment, ii) augment widespread availability of high-quality and unbiased preference studies, iii) develop quality criteria to standardize PPS conduct and evaluation, and iv) increase interactions between stakeholders on robust PPS designs (Janssens et al., 2019a; van Overbeeke et al., 2019a; Whichello et al., 2019). Stakeholders and especially regulators were concerned by a potential misuse of PPS results to overrule the traditional efficacy and safety criteria used for marketing authorization and lack of robust PPS results (Janssens et al., 2019a; van Overbeeke et al., 2019a; Whichello et al., 2019).

Other barriers highlighted by regulators and others included potential biases (in sample determination, question framing attribute selection, patient recruitment), lack of patient-centricity in PPS design, lack of patient representativity (aspects that would limit the alignment between the preference study sample and patient sample affected by the regulatory decision), lack of adequate information regarding attributes, and lack of information towards the patients as to what would the PPS be used for. In addition to the above, remaining open research questions for further methods’ research have been voiced by regulators and others in the field such as how to characterise and capture preference heterogeneity between patients. These are described in detail in the separate paper by Smith et al., *“Methodological Priorities for Patient Preferences Research: Stakeholder Input to the PREFER Public-Private Project”* (Smith et al., 2021). Numerous open questions were also raised by those interviewed and members of IMI PREFER which could not be addressed within the timeframe of the IMI PREFER project itself (Janssens et al., 2019a; van Overbeeke et al., 2019a; Whichello et al., 2019; The PREFER consortium, 2022). These questions lend themselves to further discussion and critical reflection, and may foster targeted multi-stakeholder discussions aimed at developing a policy agenda regarding future design and use of PPS:

1. What is the context of use of a PPS; i.e., what decision point(s) should be addressed and targeted in the PPS; (how) should they be designed to answer multiple decisions such as described in the PREFER recommendations and/or should they be targeted towards a particular setting such as clinical trial endpoint selection or effect size trade-offs? Two key use cases for PPS were put forward in the PREFER recommendations and endorsed in the CHMP Qualification Opinion; namely, the use of PPS to inform the selection of clinical trial endpoints and the use of PPS to identify and value trade-offs for benefits and risks (European Medicines Agency, 2021a; European Medicines Agency, 2021b). Should both aims be addressed within one PPS or should different PPS’ be designed for each research question?
2. Should a product independent broad approach to the design of PPS be taken (such as those conducted within the case studies of PREFER, patient groups, and others) and/or a product-specific, (industry) approach which is typically more compound focused?
3. How do different PPS relate to one another? E.g., when multiple PPS are being conducted in one disease area, is there a need for aggregating, summarizing, and comparing the PPS data obtained?
4. Who should optimally be financing, designing, conducting, and coordinating PPS including data governance (industry, academia, patient organizations, multi-stakeholder efforts, or public private partnerships)?
5. Which stakeholder(s) should take the lead in obtaining PPS evidence for regulatory submission and evaluation? Many stakeholders have to be involved in a collaborative effort and sponsor ownership needs to be allocated. How can the value of PPS be communicated and necessary investment secured?
6. How detailed and stringent should PPS standards be described for researchers conducting PPS and provided via guidance? And related to this, how to balance between the risk of hampering PPS research while providing enough detail towards PPS researchers?
7. Where and how to include the information in the documents used for decision making by regulators to create transparency in how the PPS has been considered (e.g., clinical trial database, regulatory assessment reports)?
8. When (within and/or outside clinical trials) to perform PPS aiming to inform these particular use cases? Is there value in doing both PPS alongside and during clinical trials? When conducted with patients participating in the clinical trial, it gives a chance to investigate their experiences and trade-offs with the treatment being investigated (i.e., revealed preferences). PPS provide also valuable results when being conducted separately from clinical trials (such studies have been conducted as part of PREFER) as trial participants often do not reflect the entire patient population.
9. How can PPS be concretely applied/operationalized aligning regulatory decisions with patient preferences? How (which methods) to include patient preferences in the regulatory process?
  • Qualitatively, taking PPS as another source of evidence; comparing evidence from PPS against clinical trial endpoints; qualitative assessment of PPS could aim to understand: i) where relevant outcomes included in clinical trial, ii) how does treatment perform against relevant outcomes?
  • Quantitatively, is there a need for application of structured methodologies such as multiple criteria decision analysis (MCDA) where patients’ values are quantitatively combined with regulators values? How can the EMA’s benefit-risk methodology (European Medicines Agency, 2023b) and FDA efforts be informative in this regard?
10. How to increase stakeholder and patient involvement in PPS design and conduct; specifically in identifying preference sensitive situations and attributes relevant for regulatory decisions?
11. How should early scientific advice/early interaction between sponsor and regulators be optimally organized in designing the PPS?
12. How could the current benefit-risk assessment (e.g., the effects table) be tailored towards allowing the incorporation of patient perspectives of relevant attributes (as revealed in PPS)?
13. How can a checklist for evaluating quality of PPS in regulatory decision-making (similar to the patient experience data checklist by FDA) be operationally implemented?
14. What is needed to ensure PPS evaluation can be transparently reported in clinical trial and regulatory documents (e.g., clinical trial database, regulatory assessment reports)?
15. Will PPS results be accepted by regulators in informing their decision making? How can early scientific advice, existing PPS guidance and PREFER recommendations be useful to address uncertainties regarding regulatory acceptance, and how can these inform detailed guidance such as the ICH guidelines by regulators that would become available at a later time point (The International Council for Harmonisation of Technical Requirements for Pharmaceuticals for Human Use ICH, 2020)? The CHMP Qualification Opinion outlines that PPS will be evaluated on a case-by-case basis and use of the PREFER framework to guide the PPS study design does not pre-empt a regulatory decision.

## 4 Discussion

This paper summarizes stakeholder-informed insights and recommendations from IMI PREFER on how PPS data can be structurally included and communicated in regulatory decision-making. Concrete PPS applications may foster discussion among regulators and others (industry, researchers, and patient organizations) on the desired context(s) of use for PPS and inform the development of regulatory guidelines (e.g., by ICH) highlighting the role of PPS in regulatory evaluation. Recommendations on when and how to consult with regulators may inform relevant discussion formats (such as scientific advice/joint consultations) on appropriate PPS research designs and may thereby lead to PPS that would be considered impactful in regulatory evaluation. Barriers and open questions related to PPS usage in regulatory evaluation lend themselves for future policy and research regarding appropriate design and use of PPS.

### 4.1 What is next for patient preference studies in regulatory evaluation?

#### 4.1.1 A call for more patient preference study use cases (pilots), guidelines and (financial) incentives

The patient preference research field seems to be dealing with the issue of “who needs to take the next step”. Is it pharmaceutical or medical device industry who should invest in PPS and include these data in their product dossiers for regulatory evaluation, or should regulators first take a more pro-active stance (e.g., by formal guidance clarifying the value, role and application of PPS in regulatory evaluation) before pharmaceutical industry will do so? Regulators point to the need for drug developers and academia to generate and submit more preference evidence (The PREFER consortium, 2022; Janssens et al., 2019a; van Overbeeke et al., 2019a; Whichello et al., 2019; European Medicines Agency, 2022b; The PREFER consortium, 2022). On the other hand, there is the acknowledgement that for these stakeholders to conduct preference studies, the regulatory environment needs to provide clarity on if and how this data will contribute to regulatory and HTA decision-making, in order for these stakeholders to do so (Janssens et al., 2019a; van Overbeeke et al., 2019a; Whichello et al., 2019; European Medicines Agency, 2022b; The PREFER consortium, 2022).

More case studies (PPS pilots) exemplifying regulatory acceptance (including how submitted PPS contributed to various decisions, and in what situations it did not and why not) could help bridge this gap. Aside from drug developers, academic researchers and clinicians, patients and patients’ organizations themselves are important stakeholders that should be incentivized and educated about preference studies so they can take up an active role in the conduct and implementation of PPS. Such PPS need to be robust and unbiased given that several regulators included in the PREFER studies expressed the fear of preference studies being designed towards meeting commercial rather than patient needs (Janssens et al., 2019a; van Overbeeke et al., 2019a; Whichello et al., 2019). PPS also need to be reported transparently towards regulators so they can assess the quality and inform the regulatory approval on a case-by-case basis. The PREFER qualified framework and the proposed topics for scientific advice outlined in Section 3.2 could inform the design of such PPS and the regulatory evaluation thereof.

Regulatory guidelines that include patient preferences as well as other forms of patient experience data could help provide incentives, acceptance, and increase (methodological) knowledge among regulators and others. Such guidelines should specify the particular value and application of preference studies in clinical trial design and regulatory evaluation and supporting documents (such as, for EMA-assessed products, in the Day 80 report, within the EPAR, in the clinical overview, or in the effects table). Guidelines should likely also describe which steps or aspect of the regulatory assessment preference studies would particularly contribute; detailing how results from PPS and conventional clinical trial outcomes, benefits, and risks would be together assessed and how they would be useful in determining clinical benefit and in establishing the overall benefit-risk balance. Guidelines also need to reflect on the complementarity of different types of patient experience data; including how the assessment of PPS alongside other forms of patient evidence such as PROs and *ad hoc* patient consultations. Guidelines may also describe how preference data would be provided in marketing application and then included in the regulatory assessment report and resulting SmPC. Guidelines should also facilitate regulators in their quality assessment of PPS; including quality criteria and concrete questions that regulators should answer to inform their assessment of the PPS. It will be important to balance the level of detail and stringency of the guidelines against the risk of stopping the preference research; the idea should be to encourage conduct and experience of PPS and formulating criteria that are too detailed may scare preference research and others less familiarized with PPS.

Beyond guidelines, other incentives are also needed to move away from the “impasse” the preference field is currently in. Should PPS be made a requirement (in some cases)? Currently, there is only requirement in the EU to include patient input on the package leaflet, but not the requirement to systematically include PPS data and/or PROs. There is also a need to better understand and create transparency regarding the impact and benefit of including preference data in clinical trial and regulatory decisions in order for stakeholders to invest in PPS. Other incentives can be developed at the level of the European Commission, in close consultation with HTA bodies (e.g., EUnetHTA) and with the support of industry, such as the European Federation of Pharmaceutical Industries and Associations (EFPIA). Financial incentives can be established via providing grants to multi-stakeholder independent public private partnerships such as a follow-on project of IMI PREFEER. These incentives should follow equity principles (as also highlighted in PREFER recommendations) and allow for structural financing of organizations across disease/health areas and stakeholders collaborating on PPS designs and conduct (incl. patient organizations, individual patients, researchers and clinicians).

There is also the question of stakeholder roles in PPS including who should financially support PPS and who should be taking the lead in conducting PPS for regulatory approval. Are PPS welcomed irrespective of what type of organization has taken the lead in designing and executing them? Does it matter whether PPS are being led by non-profit or academic institutes, patient organizations, pharmaceutical/medical device industry? Likely PPS are best conducted by multi-stakeholder partnerships (such as within IMI PREFER) but which party should take the lead in setting the research questions, deciding on the attributes to be included, analysing, and managing the PPS data and communicating these to external stakeholders? These questions should be prioritized in next discussions.

#### 4.1.2 More cross-stakeholder collaborations to increase regulatory familiarity and experience with patient preference studies

Regulators (among other stakeholders) lack familiarity and experience with respect to the methodologies to conduct preference studies, as well the ways to evaluate preference studies (Janssens et al., 2019a; van Overbeeke et al., 2019a; Whichello et al., 2019). This triggers the question of how to interact with regulators on preference study designs if they are not familiar with preference study methods. The PREFER EMA qualification procedure and the inclusion of regulatory exchanges in the context of the PREFER trajectory were useful ways to increase knowledge during the PREFER projects’ trajectory and such discussions that encourage engagement between assessors, applicants and patients should be continued following the end of the PREFER project. PPS pilot projects, webinars, multi-stakeholder workshops [such as the recent EMA workshop on patient experience data (European Medicines Agency, 2022b)] should be continuously organized to keep the momentum and exchange latest insights on PPS conduct and application. Including scientific PPS expertise within the regulatory assessment as well as increasing opportunities for discussions, workshops, and educational activities aiming to increase familiarity among regulators and others should hence be useful in this regard.

### 4.2 Limitations and steps forward to address these

The PREFER recommendations and findings presented in this paper are based upon extensive literature reviews and stakeholder studies, and a formal qualification procedure that included public consultation. However, given the qualitative nature of many of the PREFER studies and activities underlying the recommendations and findings presented in this paper, there are stakeholders whose views we have missed; potentially those stakeholders less interested and motivated to discuss patient preferences. Future activities should focus on bringing together stakeholders, including regulators, with different levels of interest and knowledge, e.g., in workshop formats and discuss on this topic. Further evaluation by stakeholders working on preference methodologies and use, as well as by clinicians and patients’ organizations would also be useful, e.g., via ongoing efforts such as guideline work ongoing by regulators and other societies.

To increase chance of adoption, the IMI PREFER recommendations on regulatory applications may suggest further inclusion among the wider healthcare community, including patient representatives, researchers experienced with qualitative and quantitative (preference) methods (e.g., academia, The Professional Society for Health Economics and Outcomes Research (ISPOR)), regulatory agencies across the globe, clinicians, HTA bodies, and payers. This will further increase their value for decision-makers and enable supplementing these insights and recommendations with other preference study experiences, evidence, and methods; for example, by adding on the experiences from applying other preference study groups or consortia outside PREFER (PREFER, 2023). The preference research field is evolving at a fast pace, and insights regarding the application and assessment of PPS in regulatory evaluation are emerging quickly, e.g., beyond IMI PREFER, the International Academy of Health Preference Research (IAHPR), and ISPOR (I AHPR, 2023; ISPOR, 2023). Combining and comparing experiences and methodological understanding from different approaches will be useful to inform the development of a standardized approach by all stakeholders across disease areas (PREFER, 2020; The International Council for Harmonisation of Technical Requirements for Pharmaceuticals for Human Use ICH, 2020). Seeking further consensus on the recommendations and concrete applications provided has the potential of rendering drug development and healthcare decision-making more patient-centered and sustainable and in the end, improve health outcomes for patients.

## 5 Conclusion and call to action

Regulatory bodies will have a leading and determining role in the future use of PPS in drug decision-making across the treatment life cycle and by all stakeholders involved. Future efforts will need to focus on creating examples of PPS use in regulatory context and development of regulatory guidelines such as those by the ICH. Regulatory guidelines detailing the design, conduct, and concrete use of preference studies in decision-making, processes for integrating PPS results in the marketing application and regulatory review process, as well as high-quality preference study case examples and demonstrated use of preference studies in drug development and evaluation will reduce uncertainty and incentivize stakeholders to systematically conduct and incorporate preference studies. Complementary to this, educational activities and multi-stakeholder discussions among international regulators, payers, academics, and patient organizations could further contribute to establish more knowledge among regulators and other stakeholders involved.

## Data Availability

The data are not publicly available because they contain information that could compromise interviewees’ privacy and consent. Requests to access these datasets should be directed to rosanne.janssens@kuleuven.be.

## References

[B1] Bouvy J. C. Cowie L. Lovett R. Morrison D. Livingstone H. Crabb N. (2020). Use of patient preference studies in HTA decision making: a nice perspective. Patient 13 (2), 145–149. 10.1007/s40271-019-00408-4 31942698

[B77] Bridges J. F. P. de Bekker-Grob E. W. Hauber B. Heidenreich S. Janssen E. Bast A. (2023). A roadmap for increasing the usefulness and impact of patient-preference studies in decision making in health: a good practices report of an ispor task force. Value Health 26 (2), 153–162. 10.1016/j.jval.2022.12.004 36754539

[B2] Brooker A. S. Carcone S. Witteman W. Krahn M. (2013). Quantitative patient preference evidence for health technology assessment: a case study. Int. J. Technol. Assess. Health Care 29 (3), 290–300. 10.1017/S0266462313000329 23863189

[B3] Churruca K. Pomare C. Ellis L. A. Long J. C. Henderson S. B. Murphy L. E. D. (2021). Patient-reported outcome measures (PROMs): a review of generic and condition-specific measures and a discussion of trends and issues. Health Expect. 24 (4), 1015–1024. 10.1111/hex.13254 33949755 PMC8369118

[B4] Cook N. S. Cave J. Holtorf A-P. (2019). Patient preference studies during early drug development: aligning stakeholders to ensure development plans meet patient needs. Front. Med. 6, 82. 10.3389/fmed.2019.00082 PMC6491461 31069227

[B5] Craig B. M. Lancsar E. Muhlbacher A. C. Brown D. S. Ostermann J. (2017). Health preference research: an overview. Patient 10 (4), 507–510. 10.1007/s40271-017-0253-9 28597377

[B6] Danner M. Hummel J. M. Volz F. Van Manen J. G. Wiegard B. Dintsios C. M. (2011). Integrating patients' views into health technology assessment: analytic hierarchy process (AHP) as a method to elicit patient preferences. Int. J. Technol. Assess. Health Care 27 (4), 369–375. 10.1017/S0266462311000523 22004779

[B7] DIA/IMI PREFER (2023). Patient preferences workshop 2021. Available at: https://www.diaglobal.org/en/conference-listing/meetings/2021/06/dia-imi-prefer-patient-preferences-workshop?utm_source=twitter&utm_medium=socialmedia&utm_campaign=21135&utm_content=page_post.

[B8] Dirksen C. D. (2014). The use of research evidence on patient preferences in health care decision-making: issues, controversies and moving forward. Expert Rev. pharmacoeconomics outcomes Res. 14 (6), 785–794. 10.1586/14737167.2014.948852 25135194

[B9] Durosini I. Janssens R. Arnou R. Veldwijk J. Smith M. Y. Monzani D. (2021). Patient preferences for lung cancer treatment: a qualitative study protocol among advanced lung cancer patients. Front. Public Health 9 (27), 622154. 10.3389/fpubh.2021.622154 33634069 PMC7900128

[B10] Egbrink M. Ijzerman M. (2014). The value of quantitative patient preferences in regulatory benefit-risk assessment. J. Mark. Access & Health Policy 2, 22761–22764. 10.3402/jmahp.v2.22761 PMC4865769 27226836

[B11] European Medicines Agency (2023b). Benefit-risk methodology. Available at: https://www.ema.europa.eu/en/about-us/what-we-do/regulatory-science-research/benefit-risk-methodology.

[B12] European Medicines Agency (2021c). CHMP & EUnetHTA parallel Scientific Advice: qualification of a Framework and “Points to consider” for method selection along with five methods for performing patient preference studies to inform regulatory and HTAbody medical product decision-making. Available at: https://www.ema.europa.eu/en/documents/regulatory-procedural-guideline/chmp-eunethta-parallel-scientific-advice-qualification-framework-points-consider-method-selection_en.pdf.

[B13] European Medicines Agency (2023a). EMA regulatory science to 2025 United Kingdom2018. Available at: https://www.ema.europa.eu/en/documents/regulatory-procedural-guideline/ema-regulatory-science-2025-strategic-reflection_en.pdf.

[B14] European Medicines Agency (2021b). IMI PREFER qualification procedure. Available at: https://www.ema.europa.eu/en/human-regulatory/research-development/scientific-advice-protocol-assistance/novel-methodologies-biomarkers/opinions-letters-support-qualification-novel-methodologies-medicine-development#imi-prefer-section.

[B15] European Medicines Agency (2022b). Multi-stakeholder workshop: patient experience data in medicines development and regulatory decision-making. Available at: https://www.ema.europa.eu/en/events/multi-stakeholder-workshop-patient-experience-data-medicines-development-regulatory-decision-making.

[B16] European Medicines Agency (2022a). Patients and consumers. Available at: https://www.ema.europa.eu/en/partners-networks/patients-consumers.

[B17] European Medicines Agency (2020). Qualification of novel methodologies for drug development: guidance to applicants 2020. Available at https://www.ema.europa.eu/en/documents/regulatory-procedural-guideline/qualification-novel-methodologies-drug-development-guidance-applicants_en.pdf.

[B18] European Medicines Agency (2021a). Qualification opinion of IMI PREFER. Available at: https://www.ema.europa.eu/en/documents/regulatory-procedural-guideline/qualification-opinion-imi-prefer_en.pdf.

[B19] European Medicines Agency (2022c). Qualification opinion of IMI PREFER. Available at: https://www.ema.europa.eu/en/documents/regulatory-procedural-guideline/qualification-opinion-imi-prefer_en.pdf.

[B20] Food and Drug Administration (2021). Pdufa reauthorization performance goals and procedures fiscal years 2023 through 2027. Available at: https://www.fda.gov/media/151712/download.

[B21] Greco M. Bere N. (2020). Patients’ emotions matter in the regulation of medicines. Available at: https://blogs.bmj.com/bmj/2020/08/11/patients-emotions-matter-in-the-regulation-of-medicines/.

[B22] Hauber B. Fairchild A. O. Reed Johnson F. (2013). Quantifying benefit-risk preferences for medical interventions: an overview of a growing empirical literature. Appl. health Econ. health policy 11 (4), 319–329. 10.1007/s40258-013-0028-y 23637054

[B23] Hines P. A. Gonzalez-Quevedo R. Lambert A. Janssens R. Freischem B. Torren Edo J. (2020b). Regulatory science to 2025: an analysis of stakeholder responses to the European medicines agency's strategy. Front. Med. (Lausanne) 7, 508. 10.3389/fmed.2020.00508 33072771 PMC7540226

[B24] Hines P. A. Janssens R. Gonzalez-Quevedo R. Lambert A. Humphreys A. J. (2020a). A future for regulatory science in the European union: the European medicines agency's strategy. Nat. Rev. Drug Discov. 19 (5), 293–294. 10.1038/d41573-020-00032-0 32235873

[B25] Ho M. P. Gonzalez J. M. Lerner H. P. Neuland C. Y. Whang J. M. McMurry-Heath M. (2015a). Incorporating patient-preference evidence into regulatory decision making. Surg. Endosc. 29 (10), 2984–2993. 10.1007/s00464-014-4044-2 25552232

[B26] Ho M. P. Gonzalez J. M. Lerner H. P. Neuland C. Y. Whang J. M. McMurry-Heath M. (2015b). Incorporating patient-preference evidence into regulatory decision making. Surg. Endosc. Other Interventional Tech. 29 (10), 2984–2993. 10.1007/s00464-014-4044-2 25552232

[B27] IAHPR (2023). International Academy of health preference research. Home page. http://iahpr.org/.

[B28] IMI PREFER (2021). CHMP & EUnetHTA parallel Scientific Advice: qualification of a Framework and “Points to consider” for method selection along with five methods for performing patient preference studies to inform regulatory and HTAbody medical product decision-making 2021. Available at: https://www.ema.europa.eu/en/documents/regulatory-procedural-guideline/chmp-eunethta-parallel-scientific-advice-qualification-framework-points-consider-method-selection_en.pdf.

[B29] Institute for Quality and Efficiency in Health Care (2014). “Choice-based Conjoint Analysis – pilot project to identify, weight, and prioritize multiple attributes in the indication “hepatitis C”,”. Report No.: GA10-03. 27905795

[B30] ISPOR (2023). Using patient preferences to inform decision making - task force. Available at: https://www.ispor.org/member-groups/task-forces/measuring-patient-preferences-to-inform-decision-making-in-health.

[B31] Janssens R. Lang T. Vallejo A. Galinsky J. Morgan K. Plate A. (2022a). What matters most to patients with multiple myeloma? A pan-European patient preference study. Front. Oncol. 12, 1027353. 10.3389/fonc.2022.1027353 36523996 PMC9745810

[B32] Janssens R. Huys I. van Overbeeke E. Whichello C. Harding S. Kübler J. (2019b). Opportunities and challenges for the inclusion of patient preferences in the medical product life cycle: a systematic review. BMC Med. Inf. Decis. Mak. 19, 189. 10.1186/s12911-019-0875-z PMC6778383 31585538

[B33] Janssens R. Huys I. van Overbeeke E. Whichello C. Harding S. Kübler J. (2019c). Opportunities and challenges for the inclusion of patient preferences in the medical product life cycle: a systematic review. BMC Med. Inf. Decis. Mak. 19 (1), 189. 10.1186/s12911-019-0875-z PMC6778383 31585538

[B34] Janssens R. Lang T. Vallejo A. Galinsky J. Plate A. Morgan K. (2021). Patient preferences for multiple myeloma treatments: a multinational qualitative study. Front. Med. 8 (930), 686165. 10.3389/fmed.2021.686165 PMC8289885 34295912

[B35] Janssens R. Pinoy J. Postmus D. Stevens H. Simoens S. Huys I. (2022b). Patient preference studies in drug development and evaluation: views from European drug regulators. Brussels: DIA EUROPE.

[B36] Janssens R. Russo S. van Overbeeke E. Whichello C. Harding S. Kubler J. (2019a). Patient preferences in the medical product life cycle: what do stakeholders think? Semi-structured qualitative interviews in europe and the USA. Patient 12, 513–526. 10.1007/s40271-019-00367-w 31222436 PMC6697755

[B37] Jimenez-Moreno A. C. van Overbeeke E. Pinto C. A. Smith I. Sharpe J. Ormrod J. (2021). Patient preferences in rare diseases: a qualitative study in neuromuscular disorders to inform a quantitative preference study. Patient 14 (5), 601–612. 10.1007/s40271-020-00482-z 33660162 PMC8357717

[B38] Kohn L. Dauvrin M. Cleemput I. (2021). Patient involvement in kce research. Belgian Health Care Knowledge Centre.

[B39] Marsh K. Caro J. J. Zaiser E. Heywood J. Hamed A. (2018). PATIENT-CENTERED decision making: LESSONS from MULTI-CRITERIA decision analysis for quantifying patient preferences. Int. J. Technol. Assess. Health Care 34 (1), 105–110. 10.1017/S0266462317001118 29277175

[B40] MEDICAL DEVICE INNOVATION CONSORTIUM (MDIC) (2015). Patient centered benefit-risk project report: a framework for incorporating information on patient preferences regarding benefit and risk into regulatory assessments of new medical technology 2015. Available at: http://mdic.org/wp-content/uploads/2015/05/MDIC_PCBR_Framework_Proof5_Web.pdf.

[B41] Monzani D. Petrocchi S. Oliveri S. Veldwijk J. Janssens R. Bailo L. (2021). Patient preferences for lung cancer treatments: a study protocol for a preference survey using discrete choice experiment and swing weighting. Front. Med. 8, 689114. 10.3389/fmed.2021.689114 PMC8365300 34409049

[B42] Mott D. J. Najafzadeh M. (2016). Whose preferences should be elicited for use in health-care decision-making? A case study using anticoagulant therapy. Expert Rev. Pharmacoeconomics Outcomes Res. 16 (1), 33–39. 10.1586/14737167.2016.1115722 26560704

[B43] Mühlbacher A. C. Bridges J. F. Bethge S. Dintsios C. M. Schwalm A. Gerber-Grote A. (2017). Preferences for antiviral therapy of chronic hepatitis C: a discrete choice experiment. Eur. J. health Econ. HEPAC health Econ. Prev. Care 18 (2), 155–165. 10.1007/s10198-016-0763-8 26846922

[B44] Mühlbacher A. C. Johnson F. R. (2017). Giving patients a meaningful voice in European health technology assessments: the role of health preference research. Patient 10 (4), 527–530. 10.1007/s40271-017-0249-5 28597373

[B45] Muhlbacher A. C. Kaczynski A. (2016). Making good decisions in healthcare with multi-criteria decision analysis: the use, current research and future development of MCDA. Appl. Health Econ. Health Policy 14 (1), 29–40. 10.1007/s40258-015-0203-4 26519081

[B46] Müller M. Barbier L. Cleemput I. Strammiello V. Dickinson S. Kübler J. (2023). EMA/EUnetHTA qualification of the IMI PREFER patient preference framework and points to consider for methods selection: key experiences, outcomes, value and implicationsEMA/EUnetHTA qualification of the IMI PREFER patient preference framework and points to consider for methods selection: key experiences, outcomes, value and implications.

[B47] Myeloma U. K. (2019). Measuring Patient Preferences: an exploratory study to determine how patient preferences data could be used in health technology assessment (HTA). Available at: https://www.myeloma.org.uk/wp-content/uploads/2019/07/NICE-Patient-Preferences-Report.pdf.

[B48] Patient-Focused Drug Development (2018). “Collecting comprehensive and representative input - guidance for industry, Food and drug administration staff, and other stakeholders - GUIDANCE,” in U.S. Department of health and human Services Food and drug administration CfDEaRC (Center for Biologics Evaluation and Research).

[B49] Postmus D. Mavris M. Hillege H. L. Salmonson T. Ryll B. Plate A. (2016). Incorporating patient preferences into drug development and regulatory decision making: results from a quantitative pilot study with cancer patients, carers, and regulators. Clin. Pharmacol. Ther. 99 (5), 548–554. 10.1002/cpt.332 26715217

[B50] Postmus D. Richard S. Bere N. van Valkenhoef G. Galinsky J. Low E. (2018). Individual trade-offs between possible benefits and risks of cancer treatments: results from a stated preference study with patients with multiple myeloma. Oncologist 23 (1), 44–51. 10.1634/theoncologist.2017-0257 29079638 PMC5759823

[B51] PREFER (2020). Patient preferences. PREFER's patient input to decision making under evaluation by EMA and EUnetHTA. Available at: https://www.imi-prefer.eu/news/news-item/?tarContentId=897422.

[B52] PREFER (2023). Testing preference elicitation methods in clinical case studies. Available at: https://www.imi-prefer.eu/case-studies/.

[B53] Russo S. Jongerius C. Faccio F. Pizzoli S. F. M. Pinto C. A. Veldwijk J. (2019). Understanding patients' preferences: a systematic review of psychological instruments used in patients' preference and decision studies. Value Health 22 (4), 491–501. 10.1016/j.jval.2018.12.007 30975401

[B54] Simons G. Janssen E. M. Veldwijk J. DiSantostefano R. L. Englbrecht M. Radawski C. (2022a). Acceptable risks of treatments to prevent rheumatoid arthritis among first-degree relatives: demographic and psychological predictors of risk tolerance. RMD Open 8 (2), e002593. 10.1136/rmdopen-2022-002593 36598004 PMC9748990

[B55] Simons G. Schölin Bywall K. Englbrecht M. Johansson E. C. DiSantostefano R. L. Radawski C. (2022b). Exploring preferences of at-risk individuals for preventive treatments for rheumatoid arthritis. Scand. J. Rheumatol. 2022, 1–11. 10.1080/03009742.2022.2116805 36178461

[B56] Simons G. Veldwijk J. DiSantostefano R. L. Englbrecht M. Radawski C. Bywall K. S. (2023). Preferences for preventive treatments for rheumatoid arthritis: discrete choice survey in the UK, Germany and Romania. Rheumatol. Oxf. Engl. 62 (2), 596–605. 10.1093/rheumatology/keac397 PMC9891433 36068022

[B57] Smith I. P. DiSantostefano R. L. de Bekker-Grob E. W. Levitan B. Berlin C. Veldwijk J. (2021). Methodological Priorities for patient preferences research: stakeholder input to the PREFER public-private project. Patient 14 (5), 449–453. 10.1007/s40271-021-00502-6 33721265 PMC8357654

[B58] Soekhai V. Whichello C. Levitan B. Veldwijk J. Pinto C. A. Donkers B. (2019). Methods for exploring and eliciting patient preferences in the medical product lifecycle: a literature review. Drug Discov. Today 24 (7), 1324–1331. 10.1016/j.drudis.2019.05.001 31077814

[B59] Teixeira M. M. Borges F. C. Ferreira P. S. Rocha J. Sepodes B. Torre C. (2022). A review of patient-reported outcomes used for regulatory approval of oncology medicinal products in the European Union between 2017 and 2020. Front. Med. (Lausanne) 9, 968272. 10.3389/fmed.2022.968272 36035431 PMC9411861

[B60] The International Council for Harmonisation of Technical Requirements for Pharmaceuticals for Human Use (ICH) (2020). ICH reflection paper on proposed ICH guideline work to advance patient focused drug development, 5.

[B61] The patient's voice in the evaluation of medicines (2013). “European medicines agency, stakeholders and communication division,”. Report No.: EMA/607864/2013.

[B62] The PREFER consortium (2022). PREFER Recommendations - why, when and how to assess and use patient preferences in medical product decision-making 2022. Available at: https://zenodo.org/record/6491042#.Y4eqf-zMIZl.

[B63] US Food and Drug Administration (2020). FDA patient engagement overview. Available at: https://www.fda.gov/patients/learn-about-fda-patient-engagement/fda-patient-engagement-overview.

[B64] US Food and Drug Administration (2021). Assessment of the use of patient experience data in regulatory decision-making. Available at: https://www.fda.gov/drugs/development-approval-process-drugs/assessment-use-patient-experience-data-regulatory-decision-making.

[B65] US Food and Drug Administration (2022). List of patient preference-sensitive priority areas 2022. Available at: https://www.fda.gov/about-fda/cdrh-patient-science-and-engagement-program/list-patient-preference-sensitive-priority-areas#oncology.

[B66] US Food and Drug Administration (2020a). Patient preference information (PPI) in medical device decision-making 2020. Available at: https://www.fda.gov/about-fda/cdrh-patient-engagement/patient-preference-information-ppi-medical-device-decision-making.

[B67] US Food and Drug Administration (2016). Patient preference information – voluntary submission, review in premarket approval applications, humanitarian device exemption applications and de novo requests, and inclusion in decision summaries and device labeling.

[B68] US Food and Drug Administration (2020b). Patient preference-sensitive areas: using patient preference information in medical device evaluation.

[B69] U.S. Department of Health and Human Services Food and Drug Administration CfDaRH, Center for Biologics Evaluation and Research (2015). Patient preference information – voluntary submission, review in premarket approval applications, humanitarian device exemption applications, and de novo requests and inclusion in decision summaries and device labeling: guidance for industry, Food and drug administration staff, and other stakeholders.

[B70] van Overbeeke E. Hauber B. Michelsen S. Peerlinck K. Lambert C. Hermans C. (2021). Patient preferences for gene therapy in haemophilia: results from the PAVING threshold technique survey. Haemophilia 27 (6), 957–966. 10.1111/hae.14401 34472162 PMC9293173

[B71] van Overbeeke E. Janssens R. Whichello C. Schölin Bywall K. Sharpe J. Nikolenko N. (2019a). Design, conduct, and use of patient preference studies in the medical product life cycle: a multi-method study. Front. Pharmacol. 10, 1395. 10.3389/fphar.2019.01395 31849657 PMC6902285

[B72] van Overbeeke E. Whichello C. Janssens R. Veldwijk J. Cleemput I. Simoens S. (2019b). Factors and situations influencing the value of patient preference studies along the medical product lifecycle: a literature review. Drug Discov. Today 24 (1), 57–68. 10.1016/j.drudis.2018.09.015 30266656

[B73] van Til J. A. Ijzerman M. J. (2014). Why should regulators consider using patient preferences in benefit-risk assessment? Pharmacoeconomics 32 (1), 1–4. 10.1007/s40273-013-0118-6 24288209

[B74] Whichello C. Bywall K. S. Mauer J. Stephen W. Cleemput I. Pinto C. A. (2020a). An overview of critical decision-points in the medical product lifecycle: where to include patient preference information in the decision-making process? Health Policy 124 (12), 1325–1332. 10.1016/j.healthpol.2020.07.007 32839011

[B75] Whichello C. Levitan B. Juhaeri J. Patadia V. DiSantostefano R. Pinto C. A. (2020b). Appraising patient preference methods for decision-making in the medical product lifecycle: an empirical comparison. BMC Med. Inf. Decis. Mak. 20 (1), 114. 10.1186/s12911-020-01142-w PMC7304129 32560655

[B76] Whichello C. van Overbeeke E. Janssens R. Schölin Bywall K. Russo S. Veldwijk J. (2019). Factors and situations affecting the value of patient preference studies: semi-structured interviews in europe and the US. Front. Pharmacol. 10, 1009. 10.3389/fphar.2019.01009 31619989 PMC6759933

